# Selenium biofortification improves bioactive composition and antioxidant status in *Plantago ovata* Forsk., a medicinal plant

**DOI:** 10.1186/s41021-023-00293-2

**Published:** 2023-12-19

**Authors:** Sankalan Dey, Sarmistha Sen Raychaudhuri

**Affiliations:** Department of Biophysics, Molecular Biology and Bioinformatics, University of Calcutta, 92, APC Road, Kolkata, 700009 India

**Keywords:** *Plantago ovata*, Selenium, Biofortification, Phytoremediation, HPLC, Metallothionein

## Abstract

**Background:**

Selenium (Se) is an essential micronutrient for humans, but its deficiency as well as toxicity affects large number of people worldwide. *Plantago ovata*, a commercially important medicinal plant, is mainly cultivated in western regions of India, where elevated levels of Se have been found in soil. Thus, we evaluated the potential of Se biofortification in *P. ovata* via phytoremediation and its effect on the bioactive composition.

**Results:**

The results showed a significant alteration in various morphological and physiological parameters in a dose-dependent manner. The 10 µM Se dose improved seedling height, biomass and total chlorophyll content. There was a gradual increase in total Se content, with highest accumulation of 457.65 µg/g FW at 500 µM Se treatment. Se positively affected the antioxidative metabolism which was measured from the change in total antioxidant capacity, radical scavenging activity and *Metallothionein 2* expression. Increasing levels of Se also affected the PAL activity, total polyphenol and flavonoid content. Caffeic acid, Coumaric acid and Rutin were found to be the most abundant phenolic compounds.

**Conclusions:**

Low levels of selenium (below 50 µM) can successfully improve Se accumulation and elicit production of various polyphenols without hampering plant growth. Thus, Se fortification of *P. ovata* seedlings via phytoremediation appears to be a feasible and efficient way to enhance its nutraceutical value in dietary products.

**Supplementary Information:**

The online version contains supplementary material available at 10.1186/s41021-023-00293-2.

## Introduction

Selenium (Se) is an essential trace element that is involved in major physiological processes like redox homeostasis, thyroid metabolism and boosts immune functions in the human body [1]. It has a relatively narrow window of beneficial effect and thus, both the deficiency and toxicity are of worldwide concern [2]. Se deficiency occurs when the consumption is below the recommended dietary allowance (RDA) of 70 µg Se day^− 1^ [3]. The low concentration of Se in soil and food crops are the primary reason behind its deficiency in several countries including India, where various food grains have been found to be below optimum levels [4–6]. Se deficiency is known to cause several health issues like hyperthyroidism, Keshan disease, cancer and weakened immune system in humans [7]. Cereal grains are the primary source of Se consumption while fruits and vegetables usually contain insignificant amounts [8]. While Se concentrations in soils are typically low (0.01–2 mg/kg), they can reach as high as 1200 mg/kg in certain parts of the world [9]. Se phytoremediation can be integrated with various biofortification practices, through consumption of plants harvested from seleniferous soils while improving soil quality [10]. Thus, fortification of agronomic crops via Se supplementation using inorganic fertilizers (like sodium selenite and sodium selenate) and bioremediation (via phytoextraction) from Se- enriched soil represents a cheap and efficient strategy to improve human nutrition.

Plants are known to take up Se in the form of selenate (the most bioavailable form) using sulfate transporters which is incorporated in proteins in the form of selenocysteine and selenomethionine via sulfate assimilation pathway [11]. This allows for mobilization of Se from soil to its bioaccumulation in edible plant parts like shoot, leaves, fruits and grains. Trace concentrations of Se have been known to be necessary for normal growth and development while moderate concentrations (up to a few micromolar) can show beneficial effects by upregulating plant metabolic pathways involved in stress resistance. At these levels, it is found to improve plant tolerance to various abiotic (like heavy metal, UV light) as well as biotic (pathogen attack and herbivory) stresses [11]. Several studies have shown that under optimum concentrations, this can increase plant productivity as well as improve antioxidative metabolism via production of various secondary metabolites [12].

Medicinal plants are an emerging candidate for Se-biofortification as they are known to be a storehouse of large number of phytochemicals (like polyphenols, flavonoids, glucosinolates etc.). These compounds have a potential antibacterial, antioxidative, anticancer, cardioprotective as well as anti-inflammatory effects which can be harnessed in pharmaceutical and medical applications [13]. Recently, young seedlings (such as sprouts and microgreens, 7–10 days after seed germination) have been shown to possess higher nutritional traits, when compared to full grown plant [14]. Application of Se in medicinal plants like garlic (*Allium sativum*), Iranian Borage (*Echiuma moenum*) basil (*Ocimum basilicum*), *Plantago asiatica*, have resulted in elevated polyphenol and flavonoid content, thus, improving nutraceutical value [15–17]. Various *Plantago* species are widely used in traditional as well as modern medicine and are known to be rich in various bioactive compounds (namely, phenylpropanoid glycosides, triterpenes, phenolic acids and flavonoids). While seeds were known to improve intestinal functions, the leaves were consumed either raw (as an addition to salads) or cooked (as an addition to soups) [18]. *Plantago ovata* (*P. ovata*), also commonly known as Isabgol or psyllium, is a medicinal herb native to Asia, the Mediterranean region of Europe and North Africa. Most of the cultivation is done in India, which is also the world leader in production of psyllium husk [19]. Along with its recognized laxative property, it has shown evidence of several health benefits like lowering the risk of colon cancer, reducing blood cholesterol levels and hyperglycaemia, ulcerative colitis and treatment for constipation and irritable bowel syndrome [20]. *P. ovata*, is considered to be rich in various phytochemicals that have a powerful antioxidant activity, thus having health-promoting properties as an ideal dietary or medicinal supplement [19, 21]. It is mainly grown as a cash crop in northwest regions of India (primarily in Gujarat, Punjab and some parts of Rajasthan) where pockets of seleniferous soils (containing total Se upto 11.6 mg/kg) have been reported [22, 23].

In the present study, we conjecture an increase in Se content along with accumulation of polyphenols in *P. ovata* seedlings under varying Se treatments to achieve biofortification. Our work aims to understand the effect of Se on biomass, photosynthetic pigments, antioxidant status and polyphenol composition under varying concentrations. The findings might provide us with relevant insights into improving the nutraceutical value of Se-fortified *P. ovata* seedlings which can be grown on selenium-rich soils of northwest India, for the purpose of decontamination (via phytoextraction) from these Se-contaminated soils or water.

## Materials and methods

### Experimental design and plant growth conditions

Seeds of *P. ovata* (cultivar HI-5) were obtained from Haryana Agricultural University, Haryana, India and all experiments were conducted on the same stock of seeds. The seeds were surface sterilised for 20 min with 10% (v/v) commercial bleach (NaOCl) and then washed five times with sterile double distilled water. They were germinated and transferred to agar-sucrose media (3% sucrose (w/v) and 0.9% agar (w/v)) containing various concentration of sodium selenate (hereafter, referred as Se concentration) i.e. 0 µM, 10 µM, 50 µM, 100 µM and 500 µM. They were grown in a plant culture room at 25 °C (± 2), under relative humidity of 55–60%, and illumination of 1500 lx for 16/8–hour durations of light/dark photoperiods. Seedlings were harvested at the eighth day of treatment and various morphological, biochemical and analytical experiments were performed.

### Measurement of growth parameters and pigment contents

Morphological attributes of control and treated seedlings like root and shoot lengths were measured using a ruler. Dry biomass was weighed after oven-drying the harvested seedlings at 70 °C until constant weight. The photosynthetic pigments were determined by the method of Sestak et al. and Lichtenthaler [24, 25]. Fresh shoot samples weighing 100 mg were homogenised in 100% ice cold acetone. The homogenates were centrifuged at 8,000 g for 10 min. Total chlorophyll and carotenoids were determined by measuring the absorbance of supernatant at 662, 663, 645, 646, and 470 nm using a JASCO V-630 UV-Vis Spectrophotometer.

### Quantitative estimation of phytochemical compounds

#### Preparation of ethanolic plant extracts

Extracts were prepared by grounding 100 mg of 7 day-old *P. ovata* seedlings in 1 ml of 50% aqueous ethanol (HPLC grade, Merck, Germany) using a mortar and pestle. The mixture was then ultrasonically treated (VC 300, Vibra Cell, Sonic materials) for 20 min in ice and centrifuged at 10,000 g for 5 min. The supernatants were collected and stored at -20 °C till further analysis.

#### Total phenolic content

Total phenols were determined using the Folin–Ciocalteu method as described by Singleton et al., with minor modifications [26]. Briefly, 50 µL of plant extract, 250 µL of Folin–Ciocalteu reagent, and 750 µL of 10% sodium carbonate was combined and incubated in the dark for 30 min at room temperature. Total phenolic content was calculated using a gallic acid standard curve after measuring absorbance at 760 nm and results were presented as µg gallic acid equivalents per g of fresh weight (GAE/gm FW).

#### Total flavonoid content

The flavonoid content was determined using aluminium chloride colorimetric method by Lin and Tang [27]. A volume of 100 µL of 10% (w/v) aluminium chloride, 100 µL of 1 M potassium acetate, and 1.8 mL of deionized water were taken in an Eppendorf tube containing 1 mL of plant extract. The mixture was incubated for 40 min at room temperature, and then absorbance was measured against the blank at 415 nm. The total flavonoid content was expressed as µg rutin equivalent (RE) per g of FW by comparing with the rutin standard curve.

#### Phenolic compound analysis by high performance liquid chromatography (HPLC)

HPLC analyses were performed using a Shimadzu prominence series HPLC system (Phenomenex, Torrance, CA) equipped with a UV-Vis detector. A reversed phase Phenomenex Luna 5 C18 column (250 × 4.6 mm particle size) (Torrance, CA) was used to carry out the separation. The mobile phase was made up of two solvents: Solvent A was water, phosphoric acid, methanol (HPLC grade, Merck, Germany) in 89.7:0.3:10 (v/v/v) respectively and Solvent B was acetonitrile, all running in a gradient setting. After filtering the extracts with an 0.2-micron syringe filter (Acrodisc, India), they were stored at -20 ºC until use. Each sample was run for 20 min at a flow rate of 1 ml/min. The column was kept at a constant 25 ºC (± 2) temperature. Phenolic components were identified and quantified by comparing retention times and spectra with those of commercially available standard compounds.

#### Total antioxidant capacity

The total antioxidant capacity of samples was calculated using the phosphomolybdenum assay method developed by Prieto et al. [28]. 0.3 mL of extract was combined with 3 mL of phosphomolybdenum reagent solution (0.6 M sulphuric acid, 28 mM sodium phosphate and 4 mM ammonium molybdate). The mixture was incubated for 90 min in a boiling water bath at 95 ºC. Then, the absorbance was read at 695 nm. The ascorbic acid calibration curve was used to calculate the amount of total antioxidant activity as µg ascorbic acid equivalents (AAE) per g FW.

#### DPPH radical-scavenging activity

The method described by Brand-Williams et al., with minor modifications was used to estimate the ability of ethanolic extracts to scavenge the free radicals [29]. A reaction mixture was made by combining 2.85 mL of a freshly prepared DPPH (2,2-Diphenyl-1-picrylhydrazyl) solution (0.06 mg/ml) in methanol along with 150 µL of the plant extract and incubated for 60 min at room temperature in dark. Absorbance was measured at 517 nm against the blank. The following formulae were used to calculate the free radical scavenging potential:

$$\begin{array}{l}DPPH{\rm{ }}\ radical{\rm{ }}\ scavenging{\rm{ }}\ activity{\rm{ }}\left( \% \right){\rm{ }} = \\{\rm{ }}\left[ {\left( {{A_{control}}-{\rm{ }}{A_{sample}}} \right)/{\rm{ }}{A_{control}}} \right]{\rm{ }} \times {\rm{ }}100\end{array}$$

where A_control_ is the absorbance of the DPPH reagent without any extract and A_sample_ is the absorbance of the DPPH reagent along with each of the samples.

### Phenylalanine ammonia lyase (PAL) activity assay

*P. ovata* seedlings (0.3 g) were homogenised in 2 ml of 0.1 M sodium borate buffer (pH 8.8) using a mortar-pestle. For the enzyme extract, the homogenised samples were centrifuged (15,000 g) for 15 min at 4 °C. PAL enzyme activity was determined using the spectrophotometric method outlined by Kovácik et al. [30]. Briefly, 350 µL of enzyme extract along with 500 µL of sodium borate buffer were pre-incubated at 40 °C for 5 min, after which the reaction was started by adding 300 µL of 50 mM l-phenylalanine. After incubation at 40 °C for 60 min, 50 µL 5 N HCl was added to stop the reaction. The amount of cinnamic acid formed was determined by measuring absorbance at 275 nm with a UV-Vis spectrophotometer (UV-1800, Shimadzu). The enzyme activity was expressed as µmol trans-cinnamic acid min^− 1^ g^− 1^ FW of tissue.

### Estimation of lipid peroxidation

The extent of lipid peroxidation was estimated using the method of Heath and Packer by determining the amount of malondialdehyde (MDA), a reactive product of thiobarbituric acid (TBARs) [31]. The samples were homogenised in 1 mL of 10% trichloroacetic acid containing 0.25% thiobarbituric acid and centrifuged at 15,000 g for 10 min. The supernatant was heated at 95 °C for 30 min in a hot water bath and then immediately cooled on ice to stop the reaction. The absorbance was recorded at 532 and 600 nm (to correct for unspecific turbidity). The concentration of lipid peroxides was expressed as micromoles per g FW of the tissues using an extinction coefficient of 155 mM^− 1^ cm^− 1^.

### Gene expression analysis of *Metallothionein 2* gene

Total RNA was extracted from 100 mg shoot tissues of 7-days old seedlings using PureLink RNA Mini kit [Ambion by Life Technologies, New Delhi, India]. To determine the expression pattern of *PoMT2*, reverse transcription PCR (RT-PCR) was carried out with gene-specific primers (Additional file 1) using Qiagen One Step RT-PCR Kit (Qiagen, New Delhi, India). Each reaction mixture contained 2 µg of total RNA. The reaction conditions are shown in (Additional file 1). RT-PCR products were run on 1.5% agarose gel and visualized in BioRad gel documentation system using ethidium bromide stain. The relative mRNA expression profile was analysed densitometrically from the band intensity of the gels using ImageJ software. Expression profile was compared against endogenous control (*β-actin*).

### Selenium determination using atomic absorption spectroscopy

The method used for mineralization of the samples and subsequent determination of Se content was followed as described by Diaz-Alarcon et al. with slight modifications [32]. Briefly, 50 mg of the sample was taken to which 2 mL of 65% HNO_3_ was added and mixed thoroughly. The caps were sealed and after 30 min, 0.5 mL of 30% H_2_O_2_ were carefully added. The vessel was sealed and then the mixture was boiled gently over a water bath (90 °C) for 60-90 min (until near colourless). After the vessel had cooled down, 2 mL of concentrated 35% HCl was added to the sample and heated at 90 °C for 20 min. Se determination was carried out using SpectrAA 50 (Varian) Atomic Absorption Spectrometer.

The evaluation of the efficiency of Se biofortification in *P. ovata* was estimated using the biofortification level (BL) according to the equation:

$$BL{\rm{ }} = {\rm{ }}C1{\rm{ }}/{\rm{ }}C2$$

where C1 is the concentration of Se in Se-fortified seedling and C2 the concentration of Se in control plants [33].

The values of Se transfer factor (TF_Se_) from the media to plant shoot were also calculated, using the following formula:

$$T{F_{Se}} = {\rm{ }}{C_{Plant}}/{\rm{ }}{C_{Medium}}$$

where C stands for Se content in seedling or medium [34].

The amount of Se provided from a portion of 10 g of *P. ovata* seedlings was used to calculate the estimated dietary intake (EDI, µg day^− 1^). EDI was additionally expressed as a percentage (EDI %) of the adequate adult intake (70 µg day^− 1^) in order to determine the contribution from the Se-fortified seedlings (Puccinelli et al., 2021). The health risk index (HRI) was also determined as the ratio between the EDI and tolerable maximum intake level (300 µg day^− 1^), to evaluate the potential health risk associated with consumption of the seedlings (Puccinelli et al., 2021).

### Statistical analysis

The experimental results were represented as Mean ± SEM. Statistical analysis was conducted with SPSS Version 26.0 (SPSS Inc., Chicago, USA) for one-way analysis of variance (ANOVA) at p < 0.05 significance level. Multiple pairwise comparisons among treatment groups were analysed using Tukey’s HSD post-hoc test. Group means not sharing the same letter are significantly different at 95% confidence level. All experiments were performed in triplicate. Principal component analysis (PCA) was performed using PAST (version 4.13).

## Results

### Growth parameters and photosynthetic pigments content

**Fig. 1:**
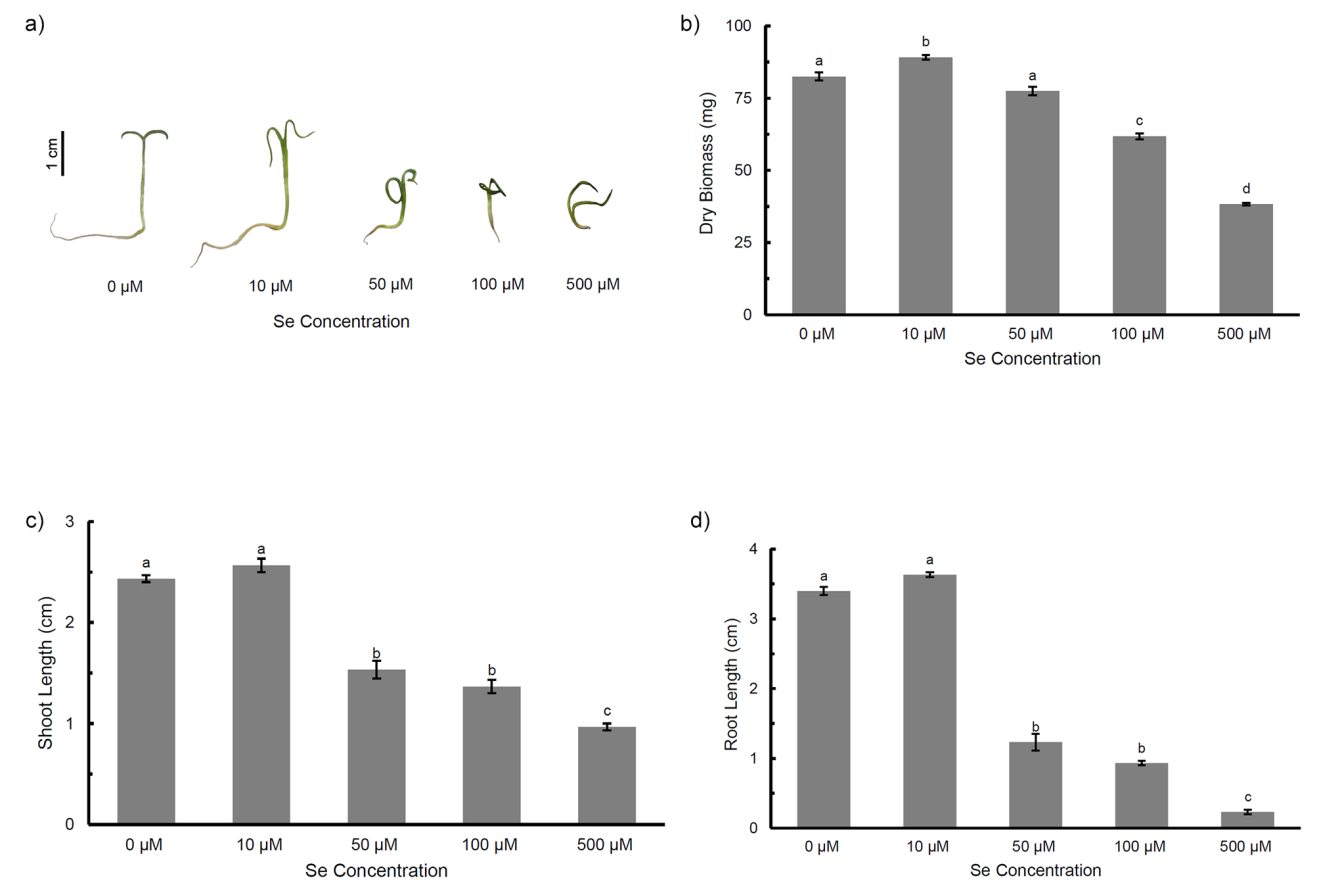
**a**) Picture of 7 days-old *Plantago ovata* seedlings **b**) Dry biomass **c**) Shoot length **d**) Root length when treated with increasing Se concentrations (Control, 10 µM, 50 µM, 100 µM and 500 µM). Verticals bars represent the mean ± SE of mean. Different letters indicate statistically significant differences between means of each treatment group (Tukey’s HSD multiple comparison at p ≤ 0.05)

**Fig. 2:**
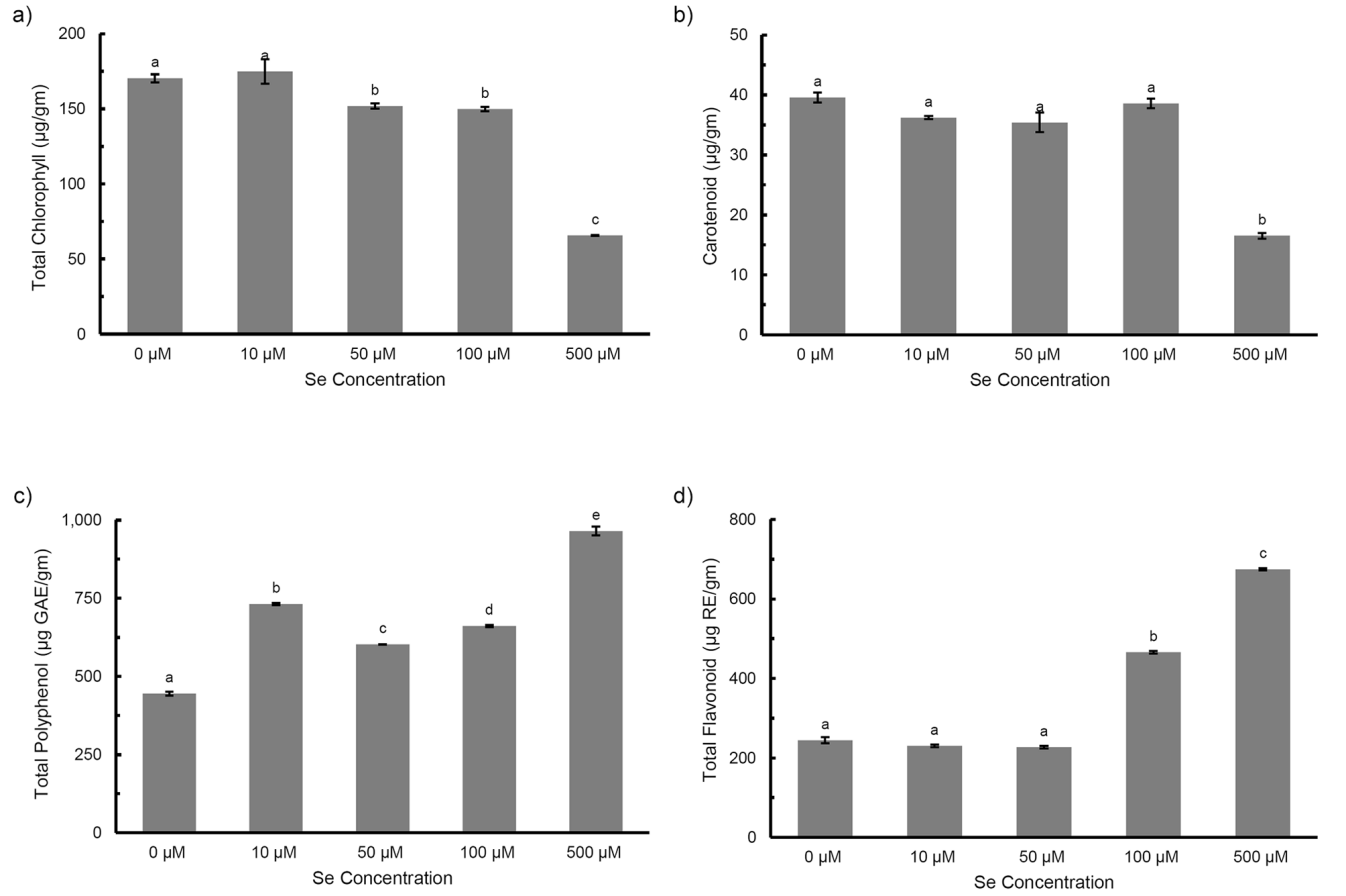
**a**) Total chlorophyll **b**) Carotenoid **c**) Total polyphenol **d**) Total flavonoid content in *Plantago ovata* seedlings subjected to increasing Se treatments. Verticals bars represent the mean ± SE of mean. Different letters indicate statistically significant differences between means of each treatment group (Tukey’s HSD multiple comparison at p ≤ 0.05)

*P. ovata* seedlings showed significant phenotypic alterations under increasing Se treatment, mainly resulting in reduction of the root and shoot length at higher concentrations. The roots were more adversely affected than the shoots (Fig. 1a). The plant dry biomass increased by 8% at low dose 10 µM Se, in comparison to 54% decrease at 500 µM Se dose (Fig. 1b). The shoot and root showed increased length at 10 µM Se, both by 6% (not significant) but eventually, decreased by 60% and 93% respectively, at higher 500 µM Se (Fig. 1c). The contents of photosynthetic pigments (total chlorophyll and carotenoid) contents in the seedlings were also observed, in which highest and lowest content of total chlorophyll was found to be in the group treated with 10 µM and 500 µM Se, respectively (Fig. 2a). Carotenoid content showed a gradual decrease with increasing Se treatment, but was not affected significantly except at high Se (500 µM) dose (Fig. 2b).

### Antioxidative metabolism

**Fig. 3:**
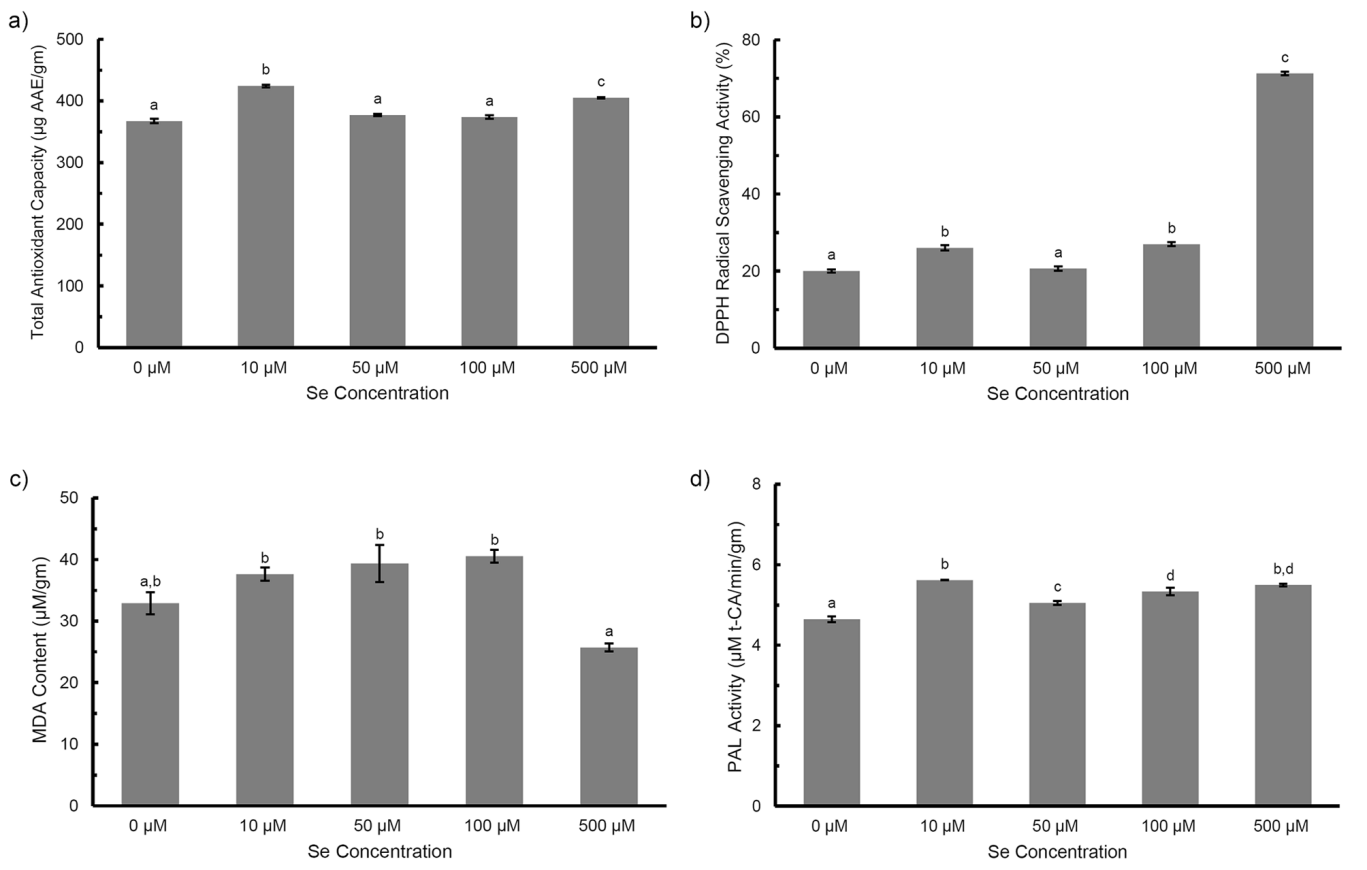
**a**) Total antioxidant capacity **b**) DPPH radical scavenging activity **c**) MDA content **d**) PAL activity in *Plantago ovata* seedlings subjected to increasing Se treatments. Verticals bars represent the mean ± SE of mean. Different letters indicate statistically significant differences between means of each treatment group (Tukey’s HSD multiple comparison at p ≤ 0.05)

The total polyphenol content (TPC) and total flavonoid content (TFC) of the seedlings increased under Se treatment. The highest increment was found to be 965.12 µg GAE/g FW (TPC) and 674.91 µg RE/g FW (TFC) when treated with 500 µM Se as compared to the untreated (Fig. 2c and d). A gradual uptrend in total antioxidant capacity (TAC) and DPPH radical scavenging activity was also recorded under increasing Se doses. Low Se dose (10 µM) induced TAC and DPPH activity by 1.2 times and 1.3 times respectively, though 500 µM showed highest radical scavenging activity (Fig. 3a and b). With increasing Se treatment, the lipid peroxidation showed slight but not significant increase in MDA content (Fig. 3c). Phenylalanine Ammonia Lyase (PAL) is a key enzyme in the phenylpropanoid pathway, which is involved in production of plant secondary metabolites. PAL activity showed significant increase with increasing Se doses but was found to be highest at 10 µM Se, in comparison to control (Fig. 3d).

### Quantitative estimation of phenolic compounds

The phenolic composition of *P. ovata* seedlings were identified and quantified using HPLC using a gradient separation. The main identified phenolic compounds were Gallic acid, Ellagic acid, Chlorogenic acid, Caffeic acid, Coumaric acid, Cinnamic acid, Ferulic acid, Vanillic acid, Syringic acid, Rutin, Quercetin and Luteolin. The most abundant phenolic compounds (µg/mg) were Caffeic acid (102.85, 328.62, 64.01, 32.28, 4.44), Coumaric acid (100.27, 147.99, 66.48, 20.03, 2.715) and Rutin (169.39, 255.23, 321.56, 133.99, 16.15) in Control, 10 µM, 50 µM, 100 µM and 500 µM respectively (Table 1). Compared to control, Ellagic acid, Chlorogenic acid, Cinnamic acid, Quercetin and Luteolin all showed a significant increase with increasing Se treatment.

**Table 1:** Quantification of important phenolic compounds in *Plantago ovata* seedlings under Se treatments

Polyphenols	Se treatments				
	0 µM	10 µM	50 µM	100 µM	500 µM
Gallic acid	1.77 ± 0.03 ^a^	1.30 ± 0.19 ^b^	1.32 ± 0.03 ^b^	0.59 ± 0.03 ^c^	0.32 ± 0.04 ^c^
Chlorogenic acid	0.39 ± 0.01 ^a^	2.41 ± 0.19 ^b^	3.98 ± 0.03 ^c^	10.49 ± 0.16 ^d^	1.15 ± 0.01 ^e^
Caffeic acid	102.85 ± 0.29 ^a^	328.62 ± 0.04 ^b^	64.01 ± 0.04 ^c^	32.28 ± 0.03 ^d^	4.44 ± 0.04 ^e^
Rutin	169.39 ± 1.95 ^a^	255.23 ± 0.19 ^b^	321.56 ± 0.04 ^c^	133.99 ± 0.03 ^d^	16.15 ± 0.30 ^e^
Coumaric acid	100.27 ± 1.76 ^a^	147.99 ± 0.20 ^b^	66.48 ± 0.01 ^c^	20.03 ± 0.26 ^d^	2.715 ± 0.04 ^e^
Quercetin	6.33 ± 0.12 ^a^	15.65 ± 0.21 ^b^	10.73 ± 0.04 ^c^	35.13 ± 0.02 ^d^	1.82 ± 0.01 ^e^
Cinnamic acid	1.42 ± 0.03 ^a^	2.35 ± 0.18 ^b^	1.98 ± 0.01 ^b^	4.57 ± 0.03 ^c^	5.25 ± 0.03 ^d^
Vanillic acid	n.d.	n.d.	0.25 ± 0.04 ^a^	0.49 ± 0.04 ^b^	0.62 ± 0.03 ^c^
Syringic acid	0.37 ± 0.01 ^a^	0.75 ± 0.29 ^a^	0.43 ± 0.02 ^a^	0.43 ± 0.05 ^a^	0.46 ± 0.01 ^a^
Ferulic acid	0.17 ± 0.00 ^a^	0.44 ± 0.04 ^b^	0.38 ± 0.03 ^b^	0.14 ± 0.06 ^a^	0.16 ± 0.03 ^a^
Luteolin	17.51 ± 0.36 ^a^	21.89 ± 0.14 ^b^	21.21 ± 0.06 ^b,d^	24.16 ± 0.07 ^c^	20.74 ± 0.03 ^d^
Ellagic acid	17.74 ± 0.23 ^a^	21.38 ± 0.18 ^b^	21.85 ± 0.03 ^b^	24.27 ± 0.08 ^c^	24.38 ± 0.03 ^c^

Quantity is expressed as ng mg^− 1^ fresh weight. n.d. – Not determined (below quantification limit). Different letters within the same row indicate statistically significant differences (one-way ANOVA followed by Tukey’s HSD multiple comparison test; P < 0.05) for each treatment group

### *Metallothionein 2* expression

**Fig. 4:**
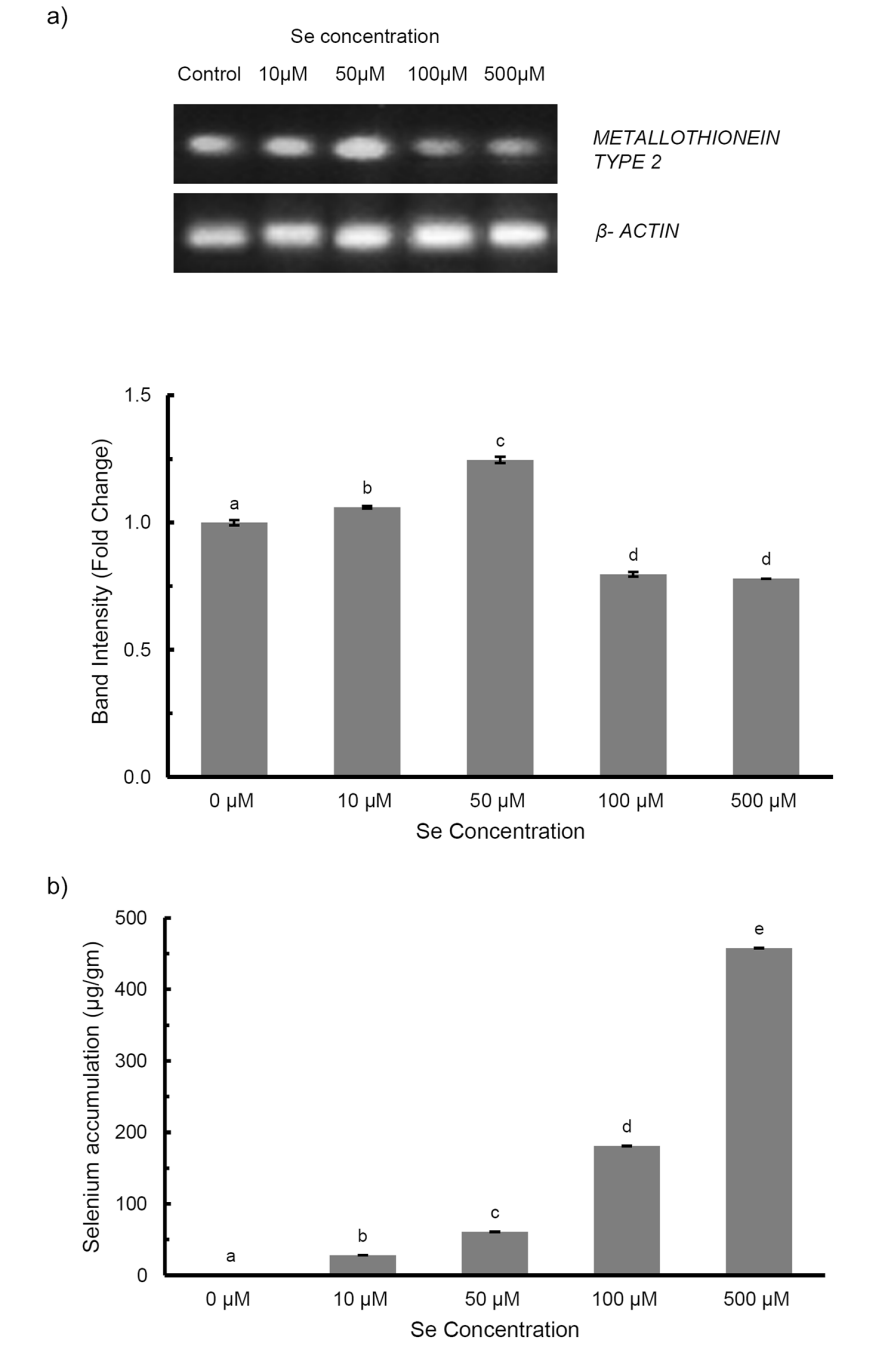
**a**) *MT2* gene expression **b**) Total Selenium accumulation in *Plantago ovata* seedlings subjected to increasing Se treatments. Verticals bars represent the mean ± SE of mean. Different letters indicate statistically significant differences between means of each treatment group (Tukey’s HSD multiple comparison at p ≤ 0.05)

Metallothionein is a low-molecular weight protein involved in binding of various heavy metals using the thiol group of its cysteine residues, which helps in protecting against metal toxicity induced oxidative stress. In our study, Se induced *PoMT2* expression till 50 µM (1.25 times) treatment which was confirmed via densitometric analysis, after which there is a decrease in the band intensity (Fig. 4a).

### Selenium content

In comparison to control, application of Se significantly increased its accumulation in *P. ovata* seedlings (Fig. 4). There was a gradual increase in Se content, from 28.18 µg/g in 10 µM till 457.65 µg/g in 500 µM Se dose. As shown in Table 2, the highest value of Se biofortification level was observed in 500 µM treatment. We also found that, the transfer factor which denotes the mobilization of Se from media, was highest at 10 µM (35.7) Se dose.

**Table 2:** Selenium biofortification level, transfer factor, EDI, HRI of *Plantago ovata* seedlings under Se treatments

Se treatments	Biofortification level	Transfer factor	EDI (µg/day)	EDI (%)	HRI
0 µM	1.00	0.00	0.56	0.80	0.0019
10 µM	52.19	35.69	29.68	42.40	0.0989
50 µM	112.73	15.42	66.17	94.53	0.2206
100 µM	335.03	22.91	179.11	255.87	0.5970
500 µM	847.62	11.59	508.56	726.51	1.6952

EDI: estimated daily intake

HRI: health risk index

The consumption of Se-fortified seedlings in reference to a portion size of 10 g (since, data concerning the average amount of daily consumption for seedlings are not available in the literature) was also calculated. The EDI ranged from 29.7 µg (10 µM) to 508.6 µg (500 µM) and will cover 42.4–736.5% of the recommended daily intake guidelines. The EDI values were below the tolerable upper intake level, i.e., 300 µg day^− 1^ till 100 µM Se, and the health risk index were below 1 (Table 2). Thus, human consumption of *P. ovata* seedlings grown below 100 µM Se levels is not expected to have a negative impact on health.

### Principal component analysis

**Fig. 5:**
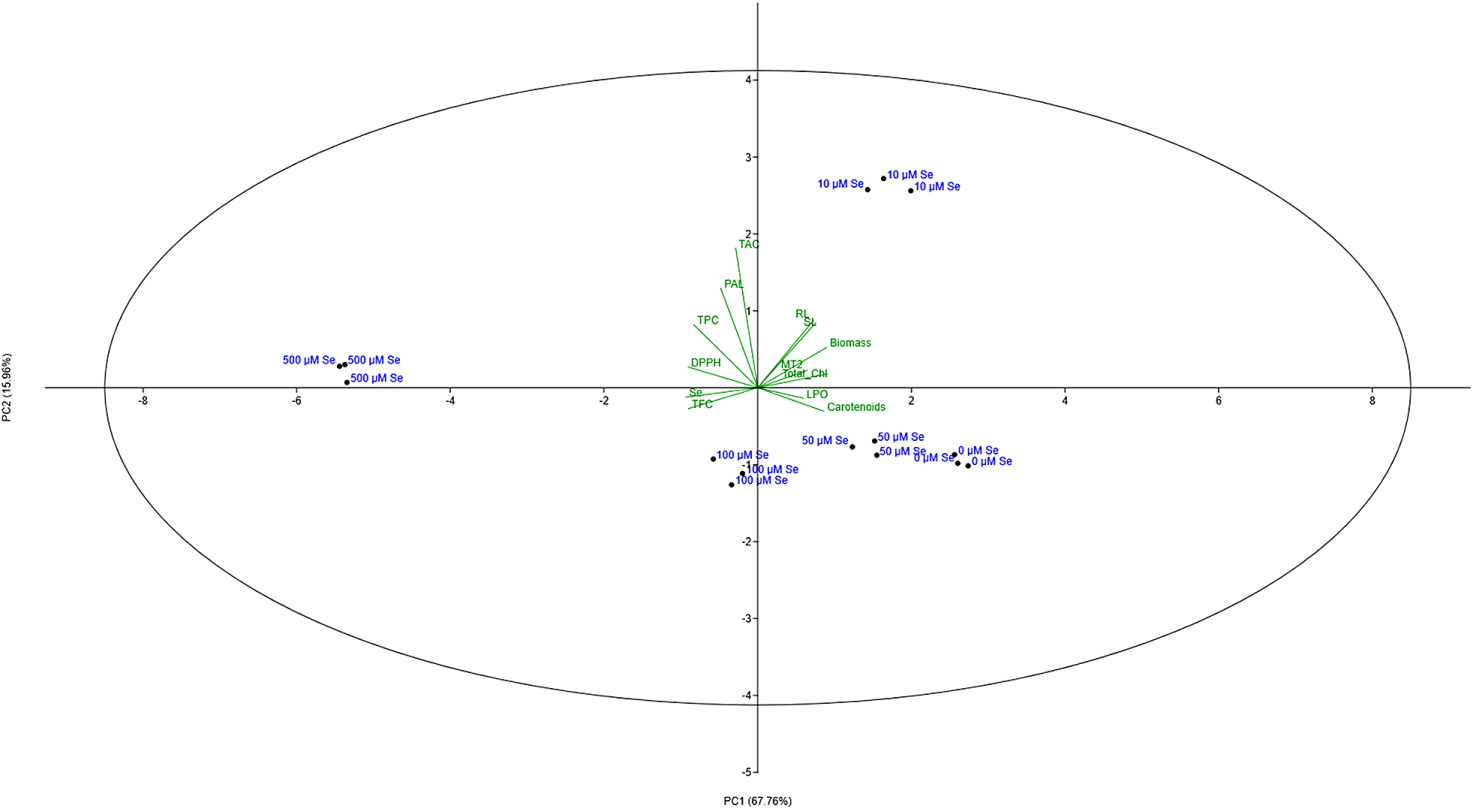
Biplot of principal component analysis of various plant parameters of *Plantago ovata* seedlings subjected to increasing Se treatments. SL: Shoot Length, RL: Root Length, Biomass: Plant dry biomass, Total_Chl: Total chlorophyll, Carotenoid: Total carotenoid content, TPC: Total polyphenol content, TFC: Total flavonoid content, TAC: Total antioxidant capacity, DPPH: DPPH radical scavenging activity, LPO: Lipid peroxidation, PAL: PAL enzyme activity, MT2: *MT2* gene expression, Se: Total Selenium content

The principal components analysis results (Fig. 5) showed PC1 and PC2 explaining 83.72% of the entire variance. PC1 (67.76%) explained the highest variation, predominantly positively correlated to shoot-root length, dry biomass and photosynthetic pigments whereas negatively correlated to TPC, TFC, DPPH and Se content. PC2 (15.96%) predominantly explained positive correlation between TAC and PAL activity. The Se treatments formed separate cluster indicating distinct response to various morphological and physiological parameters tested with TPC and TFC showing positive linear correlation with Se content.

## Discussion

Agronomic biofortification of *P. ovata* with Se is an efficient strategy to increase Se concentration as well as improving its bioactive composition. Our study showed that morphological and physiological responses of seedlings were differentially affected in a dose-dependent manner. The increase of seedling biomass and shoot-root length for Se at lowest level (i.e., 10 µM) would suggest a hormetic effect, being advantageous for plant growth [35]. While, the decrease in growth parameters at higher Se levels can be attributed to its toxic effects on *P. ovata* seedlings [36]. The results are similar to the findings of Nascimento et al. in rocket plants, where the yield maximized with 20 µM Se treatment but reduced at higher concentrations [37]. A reduction of growth for Se at higher levels was also observed for rice sprouts by D’Amato et al. [38]. The observed growth response of *P. ovata* seedlings under low Se concentrations could signify that the environmentally relevant concentrations of Se found in seleniferous soils may positively influence plant performance. Plant photosynthetic performance (inferred from total chlorophyll content) was affected at higher Se levels (50 µM and above) may be due to the degradation of porphobilinogen synthetase, required for chlorophyll biosynthesis as well as phytoene synthase (PSY), the key enzyme for carotenoid production [39, 40]. The total carotenoid content was not significantly affected by Se treatment which is in line with the findings of Chomchan et al. in ricegrass [41].

Se-biofortification is also known to influence the content of various bioactive compounds. There is growing evidence indicating the positive effects of these phytochemicals on human health. Compounds like polyphenols and flavonoids, are of most importance due to their potential role in diminishing the risk of oxidative stress-related diseases while also acting as a potent anticarcinogen [42, 43]. In our study, there was an increase in total polyphenol and flavonoid content, accumulating highest concentration at 500 µM Se treatment. Se-induced stress in seedlings triggered the production of phenolic compounds through the activation of the phenylpropanoid pathway, as evident from the PAL activity assay. Similar findings have also been reported in chickpea sprouts and wheat microgreens where PAL activity was elevated in Se-treated samples [44, 45]. With regards to individual polyphenols, the presence of various phenolic acids and flavonoids in *P. ovata* have been reported by Patel et al., but there is no evidence yet for the effect of Se on these [21]. The quantification of various polyphenols like hydroxybenzoic acid derivatives (Gallic acid, Vanillic acid, Syringic acid), Tannins (Ellagic acid), hydroxycinnamic acid derivatives (Chlorogenic acid, Caffeic acid, Coumaric acid, Cinnamic acid, Ferulic acid), Flavones (Luteolin) and Flavonols (Rutin, Quercetin), were found to be upregulated in our study. Among these, caffeic acid, coumaric acid and rutin showed the highest increase in the presence of low dose Se (10 µM, 50 µM) which play a crucial role in improving plant tolerance to temperature, salinity, drought and heavy metals stress [46].

The elevated levels of polyphenols at increasing Se levels can be explained by taking into account the production of reactive oxygen species (ROS) under Se-stress induced signalling. Plants have been known to induce the production of various antioxidants, as a protective mechanism in response to these stress which helps in ROS scavenging. This could explain the increase in both total antioxidant capacity as well as radical scavenging activity, at 10 µM Se treatment in our study. Our results are consistent with the findings of Islam et al. in wheat and Huang et al. in soybean wherein exogenous Se application at low doses resulted in elevated antioxidative status, thereby improving its stress resilience [45, 47]. We also found, the values of MDA content, a marker of lipid peroxidation status, to be consistent under increasing Se treatments. The *P. ovata* seedlings maintained cellular homeostasis and reduced the oxidative stress-induced damage, which is important for preserving cell membrane permeability [48]. Plant metallothionein proteins also play a pivotal role in providing metal tolerance by forming complexes with metal ions. In our study, *MT2* gene expression increased initially (till 50 µM dose) followed by a steady decline. Similar observations were also made by Jain et al. in sugarcane and Malik et al. in mungbean [49, 50]. These observations might imply the protective role of MT2 from toxic effects of Se when present in moderate concentrations. The decrease in expression at high Se levels might be the failure of detoxification mechanisms due to overall metabolic dysfunction.

In the current study, the treatment groups accumulated a significant amount of Se when compared to the control group. The results are in agreement with studies conducted on various microgreens such as *Ocimum basilicum*, *Coriandrum sativum*, *Brassica rapa*, *Rumex acetosa* L., *Plantago coronopus* L., and *Portulaca oleracea* demonstrating the efficacy of achieving Se-fortification in young seedlings [3, 51, 52]. Our findings show that, *P. ovata* can be a promising candidate for Se biofortification, due to its capability to grow in a Se-enriched medium with better plant biomass, rich in phenolic compounds which may be utilized as a source of dietary supplement. Also, based on the concentrations of Se found in our study, a 10 g serving in the form of sprouts or microgreens in salads, can provide upto 94.5% (at 50 µM dose) of the recommended daily Se intake, safely. The levels are well under the maximum tolerable intake level of 300 µg day ^− 1^. Thus, irrigation of *P. ovata* in Se-enriched soil is ideal for phytoremediation while immensely improving its nutraceutical value.

Our study provides a robust mechanistic insight into understanding the effects of varying Se concentrations on plant growth parameters, physiological processes as well as the altered phenolic profile in an important cash crop having immense nutraceutical value. However, it would be necessary to apply these results in field trials where plants are subjected to constantly changing environmental conditions. Also, further analytical tests to identify the accumulation of various organic forms of Se (selenocysteine and selenomethionine) with better bioavailability for animals and humans can be of eminent interest. The findings corroborate that low concentrations of Se can maximise plant yield while improving its bioactive composition. Such fortified plant products can help to maximise the therapeutic potential of antioxidant constituents in the human diet. Compounds such as caffeic acid, chlorogenic acid, quercetin, rutin and Se are of particular interest, as they have been shown to have positive effects in the limited in vitro studies done [53, 54]. Thus, agronomic practices like biofortification can help us to meet the rising demand in production of functional foods and medicinal products, while also having immense pharmaceutical relevance.

## Conclusion

The present work demonstrated the agronomic fortification of *P. ovata* seedlings using lower sodium selenate doses (below 50 µM) was ideal to trigger the synthesis of phenolic compounds without compromising growth and physiological processes. The cultivation of *P. ovata* in Se-enriched soils of western India provides a feasible and efficient way to increase the potential Se content while improving its nutraceutical value for human consumption to achieve the health benefits. Further studies should focus on phenotypes associated with quantitative traits so as to maximise plant yield as well as its stress resilience.

### Electronic supplementary material

Below is the link to the electronic supplementary material.

Supplementary Material 1

## Data Availability

The datasets used and/or analysed during the current study are available from the corresponding author on reasonable request.

## Abbreviations

DPPH 1: 1-diphenyl-2-picryl-hydrazyl
EDI: Estimated dietary intake
HPLC: High-performance liquid chromatography
HRI: Health risk index
MDA: Malondialdehyde
PAL: Phenylalanine ammonia lyase
PCA: Principal component analysis
*PoMT2*: *Plantago ovata Metallothionein 2*
RDA: Recommended dietary allowance
ROS: Reactive oxygen species
RT-PCR: Reverse transcription polymerase chain reaction
Se: Selenium
TAC: Total antioxidant capacity
TBARs: Thiobarbituric reactive substance
TFC: Total flavonoid content
TPC: Total polyphenol content
