# Supplementary material for: Selenium biofortification improves bioactive composition and antioxidant status in Plantago ovata Forsk., a medicinal plant
Source: Genes Environ. 2023 Dec 19;45:38. doi: 10.1186/s41021-023-00293-2 (PMC10729483; doi:10.1186/s41021-023-00293-2)
Supplement: Supplementary file 1 — Supplementary Material 1 [file 41021_2023_293_MOESM1_ESM.pdf]

Primer sequences and Reverse Transcription-PCR conditions

Table 1. Primer sequences of *Plantago ovata Metallothionein 2* (*PoMT2*)

Name of gene	Forward primer	Reverse primer
PoMT2	5\` ATGTCTTGCTGCAACGGAAACT 3\`	5\` CTATTTGCAATTGCATGGATTG 3\`

Table 2. Reverse transcription-PCR conditions used in the expression analysis of *PoMT2*

Reverse transcription	Temperature	50 °C	-
	Duration	30 min.	
Initial denaturation	Temperature	95 °C	-
	Duration	15 min.	
Denaturation	Temperature	94 °C	25 Cycles
	Duration	1 min.	
Annealing	Temperature	55 °C	
	Duration	30 sec.	
Extension	Temperature	72 °C	
	Duration	1 min. 30 sec.	
Final extension	Temperature	72 °C	-
	Duration	10 min.	
